# A COHORT LONGITUDINAL STUDY OF SOCIAL CAPITAL AND DEPRESSIVE SYMPTOMS IN THE WISCONSIN LONGITUDINAL STUDY

**DOI:** 10.1093/geroni/igz038.3118

**Published:** 2019-11-08

**Authors:** Kyle A Carr

**Affiliations:** Boston College, Chestnut Hill, Massachusetts, United States

## Abstract

This study examined the association between the two dimensions of social capital, structural and cognitive, and depression, as well as investigating their within- and between-effects. Using the Wisconsin Longitudinal Study, I applied a multi-level 2-wave longitudinal analysis, over a 7-year period, to examine these two dimensions of social capital influence on individual’s depressive symptoms at both the between- and within person levels. Results suggest both dimensions of social capital are negatively related with levels of depressive symptoms for individuals. The within-person changes for both self-efficacy and sense of belonging were larger than the estimates of between-effects, while trust and structural social capital effects were equal. These findings add to the growing body of literature examining depressive symptoms in late life, while also providing evidence for policymakers to hone in on key areas that can address depressive symptoms with social capital interventions.

